# The ability of branched-chain amino acid-to-tyrosine ratio (BTR) to assess preoperative liver function of patients with hepatocellular carcinoma

**DOI:** 10.1371/journal.pone.0344938

**Published:** 2026-04-02

**Authors:** Akihiko Takagi, Akitsugu Fujita, Satoshi Tokuda, Shunsuke Tamura, Eiji Nakatani, Hideyuki Kanemoto, Noriyuki Oba

**Affiliations:** 1 Department of Gastroenterological Surgery, Shizuoka General Hospital, Shizuoka, Japan; 2 Division of Statistical Analysis, Research Support, Center, Shizuoka General Hospital, Shizuoka, Japan; Nanjing University, CHINA

## Abstract

Assessment of hepatic functional reserve is critically important for preventing serious complications after hepatectomy such as liver failure. While the indocyanine green clearance test (ICG) is a liver-specific test that is not affected by other organs and is commonly used to evaluate liver reserve capacity. We lack knowledge regarding what test should be performed for patients with jaundice, portal shunts, or intolerance whose liver function cannot be accurately assessed by ICG. To close this gap, we focused on changes in amino acid metabolism associated with impaired liver function. The branched-chain amino acid-to-tyrosine ratio (BTR) reflects the severity of liver disease. The research objectives are to evaluate whether BTR is useful as an alternative test to ICG. The primary endpoint of this study is to clarify the correlation between BTR and ICG. The secondary endpoints are to provide pathological confirmation of liver fibrosis and clarify the relationship with short- and long-term outcome and to examine whether it is clinically significant as a marker of preoperative liver reserve capacity. This retrospective single-center cohort study included patients who underwent hepatectomy for HCC between January 2011 and December 2016. In this study, 235 patients were enrolled, with a median BTR of 5.58. The BTR and indocyanine green stagnation rates at 15 min (ICG-R15) showed a significant correlation (r = −0.57, p < 0.001), whereas 1/BTR showed an even stronger correlation (r = 0.66, p < 0.0001). Conversion formulas that is a regression equation predicting ICG test results with BTR as an explanatory variable were analyzed. In addition to its correlation with ICG-R15, BTR was significantly associated with liver fibrosis in the background liver pathology of resected specimens, demonstrating higher sensitivity for detecting cirrhosis compared to ICG. The high BTR group exhibited significantly longer survival than the low BTR group (p = 0.020). The results indicate that BTR and ICG are significantly correlated. Comparable to ICG, BTR is a predictor of liver fibrosis and a prognostic factor for postoperative outcomes in patients with HCC. Although 99mTc-GSA scintigraphy has been reported to correlate with IGC, the correlation coefficient and number of cases in this study are equivalent. BTR can be tested through routine blood sampling. The results of this study demonstrate the potential for clinically evaluating preoperative hepatic reserve capacity in a less invasive, more cost-effective without facility limitations.

## Introduction

Primary liver cancer is the sixth most common cancer and the third leading cause of cancer-related deaths worldwide. Hepatocellular carcinoma (HCC) accounts for 75–85% of all primary liver cancers [1]. Evaluation of liver function is important in determining treatment options for HCC [2,3]. Whether hepatectomy—the most radical treatment for HCC—is chosen, and the extent to which liver volume can be safely removed depends on liver function. The Child–Pugh classification and the degree of liver damage are typical methods for preoperative liver function assessment [4,5]. In recent years, it has been reported that the model for end-stage liver disease (MELD) [6] and the albumin–bilirubin (ALBI) scores [7], which can be evaluated solely by objective numerical values, are useful for assessing liver reserve capacity. The variables for the MELD score are Cr, Bil, and INR, while the variables for the ALBI score are ALB and Bil. These can be confirmed by a general blood test, but the effects of organ failure other than liver failure are also significant [8,9].

The indocyanine green (ICG) clearance test is one of the factors used to classify the degree of liver damage and is specific to the liver. ICG is a fluorescent dye selectively taken up by the liver and excreted in bile. The stagnation or disappearance rate, measured 15 min after intravenous injection, is calculated. If ICG is not available, an alternative method is required to evaluate liver function before resection. The Japanese guidelines for HCC recommend hepatobiliary scintigraphy with 99mTc-GSA. However, the scintigraphy is not a popular assessment tool in many institutions because of limitations on the use of the radionuclide generator 99mTc [10,11].

The branched-chain amino acid-to-tyrosine ratio (BTR) is a blood test specific to the liver that reflects liver function. To clarify why BTR reflects liver dysfunction, it is necessary to understand the physiological mechanisms of BTR. Tyrosine is metabolized mainly by tyrosine aminotransferase, which is mainly found in the liver. Therefore, the level of tyrosine in the blood rises due to impaired hepatic metabolism [12]. Furthermore, impaired hepatic metabolism reduces the ability to process ammonia through the urea cycle. This causes blood ammonia levels to rise [13]. Ammonia that escapes hepatic metabolism is processed and detoxified by glutamate in muscle. The branched-chain amino acid (BCAA) provide nitrogen to α-ketoglutarate (α-KG) in the synthesis of glutamate. Elevated ammonia increases BCAA consumption. A decrease in BCAA causes muscle catabolism, resulting in a decrease in muscle mass [14]. Under liver dysfunction, glycogen storage capacity of the liver decreases. As a result, the amount of sugar transported to the periphery increases, but sugar utilization also decreases due to a decrease in muscle mass. This increases insulin levels and insulin resistance. This condition promotes the uptake of BCAAs into the muscles, causing a decrease in BCAAs in the blood [15]. Liver dysfunction is reflected low BTR through decreased metabolic capacity, muscle catabolism, and increased insulin resistance. The BCAA to Aromatic amino acids (AAA) including tyrosine molar ratio is an indicator of reduced hepatic metabolic function, referred to as Fischer’s ratio [16]. BTR, defined as the (valine + leucine + isoleucine)/tyrosine molar ratio, differs from Fischer’s ratio in that only tyrosine is measured as AAA. BTR is a simple and cost-effective alternative to Fischer’s ratio. In Japan, BTR is commonly used to assess the amino acid imbalance. Evidence-based clinical practice guidelines for liver cirrhosis 2020 by the Japan society of hepatology [10,11], the following are recommended as nutritional assessment factors during BCAA administration. The cost of BTR testing is approximately $10, handled by multiple clinical testing companies.

Although several studies have examined the relationship between the ICG test and surgical outcomes of HCC [17–19], BTR has not been similarly investigated. There is one paper reporting on the correlation between BTR and ICG. However, the number of cases is small, with only 22 cases, and among them, only 7 cases underwent liver resection for hepatocellular carcinoma, while 8 cases were gastric cancer patients [20]. No studies have been conducted to analyze the correlation between preoperative BTR and ICG in patients with hepatocellular carcinoma undergoing liver resection, for whom liver reserve capacity should be evaluated most critically. To our knowledge, this study is the first study to examine the correlation between BTR and ICG including surgical outcomes by standardizing the target disease to hepatocellular carcinoma and the surgical procedure to liver resection.

Furthermore, no research has evaluated how accurately these two test values correlate or whether a substitutable relationship exists.

This study aimed to demonstrate the significance of BTR in hepatectomy for HCC, analyze the accuracy of the correlation between ICG and BTR, and determine whether a substitutable relationship exists.

## Methods

### Patients

This single-center, retrospective cohort study was conducted at Shizuoka General Hospital. The study protocol was approved by the hospital’s ethics committee (SGHIRB#2021050). This study was conducted in accordance with the principles of the Declaration of Helsinki. This study was a retrospective study and did not require invasive intervention. It was determined by the hospital’s ethics committee that written consent was not required. and consent was obtained through an opt-out method. The data were collected between December 2021 and March 2022 and analyzed anonymously by several physicians on the basis of medical records.

In this study, the inclusion criteria were as follows: (1) patients who under went hepatectomy for HCC at Shizuoka General Hospital between January 2011 and December 2016; (2) both BTR and ICG testing were performed preoperatively (3) hepatectomy included both primary and repeat hepatectomy. The exclusion criteria were as follow (1) Patients whose postoperative pathological diagnosis was other than hepatocellular carcinoma.

### BTR and ICG test procedure

Blood collection for the BTR test was conducted directly prior to the ICG test using routine blood tests procedure. BTR was measured by enzymatic method. The ICG test was conducted preoperatively according to the following procedure: After an overnight fast and while at rest, a dose of 50 mg ICG dissolved in 10 mL of sterile water was injected through a peripheral vein, based on the patient’s body weight (0.5 mg/kg). The 15-min retention rate of ICG (ICG-R15) and the elimination rate constant (ICG-K) were measured using a pulse spectrophotometer (DDG-3300K, Japan). Accordingly, blood samples for the BTR test were also collected in the morning under fasting conditions.

### Data collection of clinicopathological characteristics

Data were extracted from electronic medical records, including sex, age, body mass index, presence and type of chronic hepatitis, preoperative blood tests (BTR and ICG), preoperative Child–Pugh classification, the degree of liver damage, MELD and ALBI score. Surgical outcomes, pathological diagnoses of the tumor, background liver disease/fibrosis in the resected liver, and long-term outcomes were also recorded. The degree of back ground liver disease/fibrosis was assessed using F stage of the New Inuyama Classification and Fstage: F0 (no fibrosis), F1 (mild fibrosis, fibrous portal expansion), F2 (moderate fibrosis, bridging fibrosis), F3 (severe fibrosis, bridging fibrosis with distorted acinar architecture), and F4 (cirrhosis).

### Definition of postoperative complications and large ascites

Postoperative complications were defined and classified according to the Clavien–Dindo (CD) classification. Patients with CD grade III or higher (CD ≥ III) were considered to have complications. Postoperative large ascites was defined as daily postoperative ascitic fluid drainage of > 10 mL/kg of preoperative body weight from thoracic and abdominal drains.

### Cut-off values for BTR in analysis of long-term outcomes

The relationship between BTR and long-term outcomes, including overall survival (OS) and recurrence-free survival (RFS), was examined by dividing the population into high and low BTR groups. The median BTR of all patients in this study was used as the cut-off value.

### Statistical analysis

Statistical analyses were performed using JMP software (version 11.0; SAS Institute Inc., Cary, NC, USA). Continuous variables are presented as medians and ranges and were compared using the Mann–Whitney U test. Categorical variables are summarized as numbers and percentages, and were compared using Pearson’s χ^2^ tests. A *p*-value of 0.05 was considered statistically significant. OS and RFS were assessed using the Kaplan–Meier method, and comparisons were made using the log-rank test.

A post-hoc power analysis was conducted using GPower software (version 3.1.9.7) to assess the adequacy of the sample size.

## Results

### Patient characteristics

A total of 235 patients were included in this study. The clinical backgrounds, surgical outcomes, and pathological findings of the patients are summarized in Table 1. The median values for BTR, ICG-R15, and ICG-K were 5.58, 12.0, and 0.143, respectively; however, ICG-K values were not available for five patients. The ICG-K-related analyses were the median analysis of ICG-K and the correlation analysis with BTR, and only these two analyses excluded the five patients from the analysis. There are no missing data in the evaluation factors except for ICG-K. Blood test results showed no significant bias. The reference ranges for BTR and ICG-R15 are 4.99–9.45 (ratio) and ≤10%, respectively. The reference ranges for other test items are as follows: ALB 3.5–5.0 g/dL, PT-INR 0.8–1.2, PT activity 70–120%, AST 10–40 IU/L, ALT 7–56 IU/L and Total Bil 0.2–1.2 mg/dL. According to the Child–Pugh classification, 212 patients (90.2%) had grade A disease and 23 (9.8%) had grade B disease; no patients were classified as grade C. Similarly, based on the Liver Damage classification, 165 patients (70.2%) had grade A and 70 (29.8%) had grade B disease; grade C was not observed. Fourteen patients (6%) received BCAA supplementation. Although there was a bias in the number of cases, BTR was significantly lower in the group that received BCAA supplementation than in the group that did not (p = 0.045).

**Table 1:** Patient characteristics.

	N = 235
**Age (years)**	72(38-92)
**Sex (n, %)**	
**Male**	191(81.3%)
**Female**	44(18.7%)
**BMI**	23.3(14.2-32.7)
**Hepatitis (n, %)**	
**B/C/non-B non-C**	30(12.8%)/137(58.3%)/68(28.9%)
**BTR**	5.58(2.31-11.05)
**ICG-R15 (%)**	12(1.1-53.5)
**AST (IU/L)**	34(5-300)
**ALT (IU/L)**	29(4-183)
**ALB (g/dl)**	4.0(2.1-5.5)
**Total Bil (mg/dl)**	0.8(0.2-2.1)
**PT (%)**	85(33-120)
**PLT (×10**^**4**^)	14.3(3.2-65)
**Child**–**Pugh grade (n, %)**	
**A/B/C**	212(90.2%)/23(9.8%)/0(0%)
**Liver Damage grade (n, %)**	
**A/B/C**	165(70.2%)/70(29.8%)/0(0%)
**BCAA supplementation (n, %)**	14(6.0%)
**Operation time (min)**	379(111-965)
**Bleeding**	560(0-8030)
**Operation type (n, %)**	
	limited hepatectomy 123(52.3%) subsegmentectomy 12(5.1%) segmentectomy 56(23.8%) bisectionectomy 43(18.3%) trisectionectomy 1(0.4%)
**Initial hepatectomy/repeat hepatectomy (n, %)**	183(77.9%)/52(22.1%)
**Postoperative complications (CD ≥ III) (n, %)**	23(9.8%)
**Postoperative large ascites (n, %)**	29(12.3%)
**Pathological diagnosis (n, %)**	
**Number of lesion 1/2/3 or more**	166(70.6%)/31(13.2%)/38(16.2%)
**Size of lesion (cm)**	3.0(0.8-20)
**Fibrosis Stage 0/1/2/3/4 (n, %)**	19(8.0%)/27(11.5%)/53(22.6%)/33(14.0%)/103(43.3%)
**R 0/1 (n, %)**	208(88.5%)/27(11.5%)
**Stage I/II/III/IV (n, %)**	50(21.3%)/91(38.7%)/57(24.3%)/37(15.7%)

Data are presented as n (%) or median (range).

*B, hepatitis B alone; BC, hepatitis B and C.*

BMI, body mass index; BTR, branched-chain amino acid-to-tyrosine ratio; ICG-R15, 15-min retention rate of indocyanine green; AST, aspartate aminotransferase; ALT, alanine aminotransferase; ALB, albumin; Bil, bilirubin; PT, prothrombin time; PLT, platelets; CD, Clavien–Dindo classification.

### The correlation between BTR and ICG

**Fig 1:**
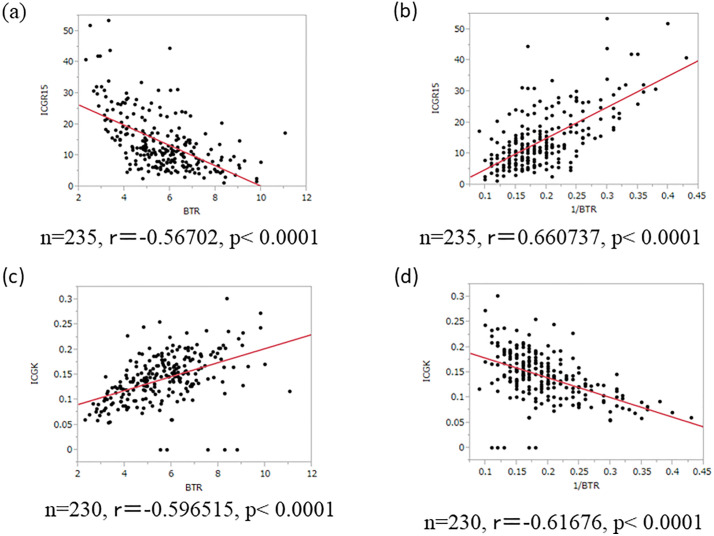
BTR and ICG. (a) Correlation between BTR and ICG-R15. (b) Correlation between 1/BTR and ICG-R15. (c) Correlation between BTR and ICG-K. (d) Correlation between BTR and ICG-K. BTR: branched-chain amino acid-to-tyrosine ratio; ICG-R15: 15-min retention rate of indocyanine green; ICG-K: elimination rate constant of indocyanine green.

A scatter plot illustrating the correlation between BTR and ICG is shown in Fig 1. BTR and ICG-R15 were significantly correlated (r = −0.57, p < 0.0001). Furthermore, the mode of distribution suggested an inversely proportional relationship. The inverse of BTR (1/BTR) and ICG-R15 showed an even stronger correlation (r = 0.66, p < 0.0001). Similarly, BTR and ICG-K were significantly correlated (r = −0.60, p < 0.0001), as were 1/BTR and ICG-K (r = −0.62, p < 0.0001).

The regression equations and correlation coefficients (r) for each combination were as follows:


ICG−R15 = 32.835611 − 3.2646081 × BTR, r = −0.57

ICG−R15 = −4.9425.5 + 100.05472 × (1/BTR), r = 0.66

ICG−K = 0.0532531 + 0.0163705 × BTR, r = 0.60

ICG−K = 0.2310455 − 0.4418465 × (1/BTR), r = −0.62

A post-hoc power analysis was conducted to assess the adequacy of the sample size. Assuming a two-sided Fisher’s z test with a significance level of 0.05, a sample size of 235 provides approximately 100% power to detect a true correlation coefficient of r = −0.57, 0.66, 0.60, −0.62. This confirms that the sample size was sufficient for detecting the observed correlation.

### BTR and MELD, BTR and ALBI

**Fig 2:**
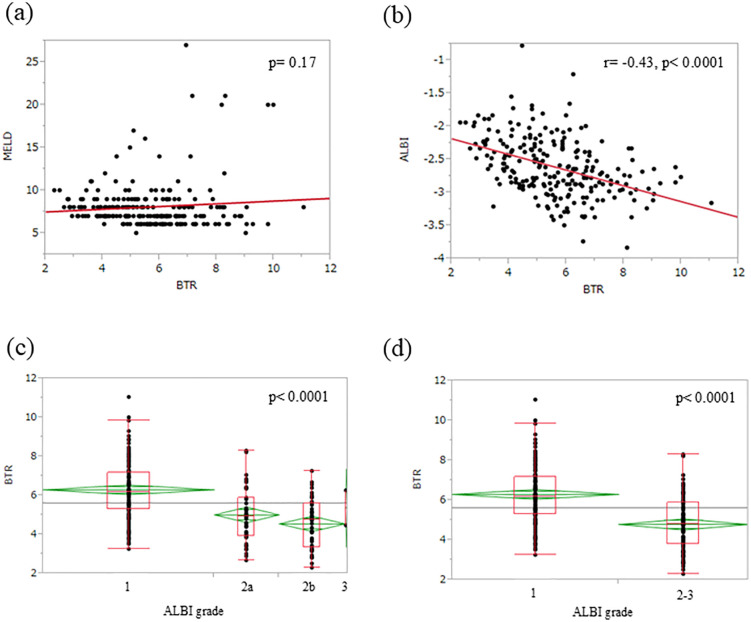
BTR and MELD and ALBI. (a) Correlation between BTR and MELD score. (b) Correlation between BTR and ALBI score. (c) BTR and each ALBI grade. (d) BTR and high or low ALBI grade (1 or 2-3).

No significant correlation was found between MELD scores and BTR (p = 0.17) (Fig 2a). A significant correlation was found between ALBI scores and BTR (r = −0.43, p < 0.0001) (Fig 2b). The number of cases and average BTR values for each ALBI grade are as follows. Grade 1 had 132 cases with an average value of 6.3, Grade 2a had 50 cases with an average value of 5.0, Grade 2b had 51 cases with an average value of 4.6, and Grade 3 had 2 cases with an average value of 5.3. Significant variation was observed in each grade (p < 0.0001) (Fig 2c). In a two-group comparison, when divided into Grade 1 and Grade 2 or higher, BTR was significantly higher in Grade 1 (p < 0.0001) (Fig 2d).

### BTR and surgical short- and long-term outcomes

Postoperative complications (CD ≥ III) were observed in 23 patients (9.8%). The types of postoperative complications are presented in Table 2. The most common complications included pleural effusion, ascites requiring puncture drainage, intra-abdominal abscesses without bile leakage, and bile leakage. One patient developed a postoperative aneurysm of the gastroepiploic artery, likely due to vascular injury caused by intraoperative manipulation.

**Table 2:** Type of post-operative complications.

	Total cases (n = 23)
**Pleural and ascites with puncture drainage**	4
**Intra-abdominal abscess (infective ascites) without bile leakage**	4
**Bile leakage**	4
**Respiratory failure**	2
**Bleeding**	2
**Aspiration pneumonia**	2
**Liver failure**	2
**aneurysm of gastroepiploic artery**	1
**Wound dehiscence**	1
**Septic shock with no identifiable cause**	1

An analysis of the association between clinicopathological factors and postoperative complications is shown in Table 3. In the univariate analysis, BTR, albumin (ALB) level, intraoperative blood loss, and operative time were significantly correlated with postoperative complications. However, none of these factors were significant independent predictors in the multivariate analysis. To assess collinearity between BTR and ALB, a correlation analysis was performed between the two variables. The correlation between BTR and ALB was significant, but the strength of the correlation was weak. (p < 0.001, r = 0.36). If there is multicollinearity between BTR and ALB, either BTR or ALBI should be included in the multivariate analysis. When ALB was selected, ALB (p = 0.020) and intraoperative blood loss (p = 0.029) were significant prognostic factors for postoperative complications. On the other hand, when BTR was selected, only intraoperative blood loss was a significant factor (p = 0.038).

**Table 3:** Analysis of association between clinicopathological factors and postoperative complications.

	Without postoperative complications	With postoperative complications	P value of univariate analysis	Odds ratio	P value of multivariate analysis
**Age (years)**	72(38-92)	75(49-87)	0.8163		
**Sex (male/female)**	172/40	19/4	0.8631		
**BMI (kg/m**^**2**^)	23.4(14.2-32.7)	23.1(16.33-29.9)	0.2981		
**HBs-Ag positive/HCV-Ab positive/non-B non-C**	25/124/63	5/13/5	0.3538		
**BTR**	5.675(2.31-11.05)	4.8(2.81-7.05)	0.0409	1.237	0.2168
**ICG-R15 (%)**	11.8(1.1-53.5)	14.9(4.8-31)	0.209		
**AST (IU/L)**	34(5-198)	37(20-300)	0.0989		
**ALT (IU/L)**	29(4-183)	31(14-156)	0.826		
**ALB (g/dl)**	4.0(2.3-5.5)	3.5(2.1-4.5)	0.0029	2.329	0.0689
**Total Bil (mg/dl)**	0.8(0.2-2.1)	0.8(0.5-2.0)	0.8979		
**PT (%)**	86(33-120)	77(66-101)	0.0766		
**PLT (×10**^**4**^)	14.6(4.6-43.6)	11.9(3.2-65)	0.5299		
**Child**–Pugh grade (A/B) **	193/19	19/4	0.1963		
Liver Damage grade (A/B) **	153/58	11/12	0.0134		
**Operation time (min)**	370.5(111-912)	473(198-965)	≤0.0001	0.998	0.3125
**Bleeding**	540(0-4970)	920(22-8030)	≤0.0001	1	0.0522
**Initial hepatectomy/repeat hepatectomy**	163/49	20/3	0.2692		

Data are presented as n (%) or median (range).

*B, hepatitis B alone; BC, hepatitis B and C.*

**There was no patient with grade C Child–Pugh classification and degree of liver damage.

BMI, body mass index; HBsAg, hepatitis B surface antigen; HCV-Ab, hepatitis C antibody; BTR, branched-chain amino acid-to-tyrosine ratio; ICG-R15, 15-min retention rate of indocyanine green; AST, aspartate aminotransferase; ALT, alanine aminotransferase; ALB, albumin; Bil, bilirubin; PT, prothrombin time; PLT, platelets.

Although the associations between BTR and each of postoperative liver failure and bile leakage were assessed, statistical significance was not demonstrated (postoperative liver failure p = 0.37, bile leakage p = 0.20).

A post-hoc power analysis was conducted to assess the adequacy of the sample size.

Assuming a multiple regression model with 4 explanatory variables, a sample size of 235 provides approximately 100% power to detect. (an effect size of f2 = 0.2178785 (adjusted coefficient of determination R2 = 0.1789).

Postoperative large ascites (> 10 mL/kg of preoperative body weight) was observed in 29 patients (12.3%). In the univariate analysis, significant factors included BTR, ICG-R15, aspartate aminotransferase and ALB levels, prothrombin time, Child–Pugh classification, degree of liver damage, operative time, and blood loss. In the multivariate analysis, the only independent predictor was operative time (Table 4).

**Table 4:** Analysis of association between clinicopathological factors and postoperative large ascites.

	Without postoperative large ascites	With postoperative large ascites	P value of univariate analysis	Odds ratio	P value of multivariate analysis
**Age (years)**	72.5(38-92)	70(49-81)	0.1822		
**Sex (male/female)**	168/38	23/6	0.7719		
**BMI (kg/m**^**2**^)	23.45(14.2-32.7)	22.1(16.33-29.9)	0.2672		
**HBs-Ag positive/HCV-Ab positive/non-B non-C**	24/119/63	6/18/5	0.1948		
**BTR**	5.755(2.31-11.05)	4.49(2.66-9.05)	0.0065	1.299	0.2184
**ICG-R15 (%)**	11.5(1.1-53.5)	14.9(2.8-43.8)	0.0041	0.996	0.9200
**AST (IU/L)**	33(5-198)	42(24-300)	0.0024	1.002	0.8159
**ALT (IU/L)**	28(4-183)	36(14-156)	0.1165		
**ALB (g/dl)**	4.0(2.3-5.5)	3.7(2.1-4.5)	0.0018	1.056	0.9229
**Total Bil (mg/dl)**	0.8(0.2-2.1)	0.8(0.4-2.0)	0.4584		
**PT (%)**	87(33-120)	76(61-103)	0.0008	1.032	0.1494
**PLT (**×**10**^**4**^)	14.6(5.0-43.6)	11.7(3.2-65)	0.3876		
**Child**–Pugh grade (A/B) **	191/15	21/8	0.0006	3.100	0.1474
Liver Damage grade (A/B) **	153/53	12/17	0.0003	1.232	0.7741
**Operation time (min)**	367.5(111-912)	502(258-965)	≤0.0001	0.994	0.0023
**Bleeding**	515(0-4970)	1430(100-8030)	≤0.0001	1.000	0.3538
**Initial hepatectomy/repeat hepatectomy**	158/48	25/4	0.2482		

Data are presented as n (%) or median (range).

*B, hepatitis B alone; BC, hepatitis B and C.*

**There was no patient with grade C Child–Pugh classification and degree of liver damage.

BMI, body mass index; HBsAg, hepatitis B surface antigen; HCV-Ab, hepatitis C antibody; BTR, branched-chain amino acid-to-tyrosine ratio; ICG-R15, 15-minretention rate of indocyanine green; AST, aspartate aminotransferase; ALT, alanine aminotransferase; ALB, albumin; Bil, bilirubin; PT, prothrombin time; PLT, platelets.

**Fig 3:**
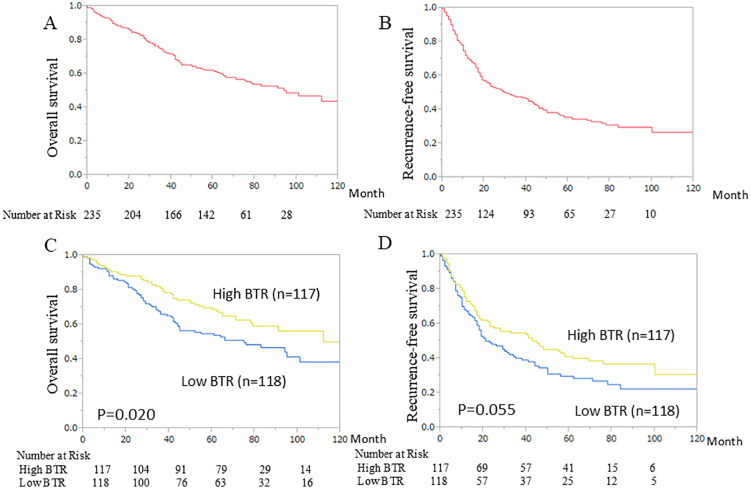
Kaplan–Meier curves for (A) overall survival (OS) and (B) recurrence–free survival (RFS) of all patients, (C) OS and (D) RFS of the two groups. High BTR (n = 117) and low BTR (n = 118) groups at a cut-off value of 5.58 (overall median) in hepatocellular carcinoma patients who underwent hepatectomy. BTR, branched-chain amino acid-to-tyrosine ratio.

**Fig 4:**
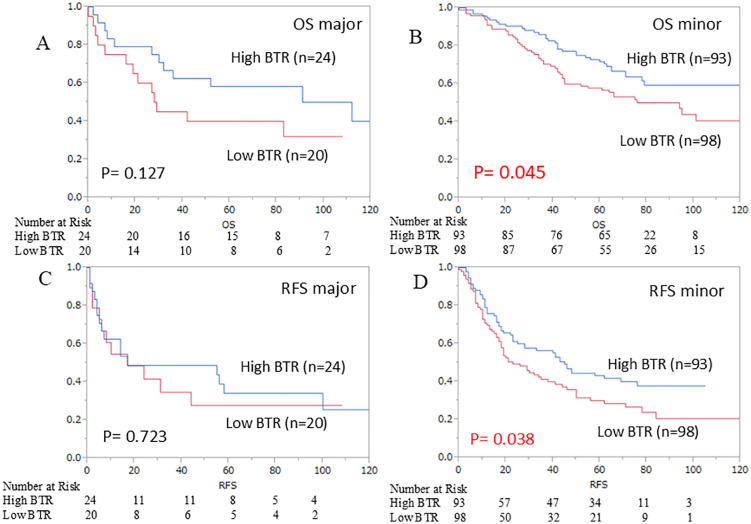
Kaplan–Meier curves for (A) overall survival (OS) of major hepatectomy group, (B) OS of minor hepatectomy group, (C) recurrence‐free survival (RFS) of major hepatectomy group (D) RFS of minor hepatectomy group.

**Fig 5:**
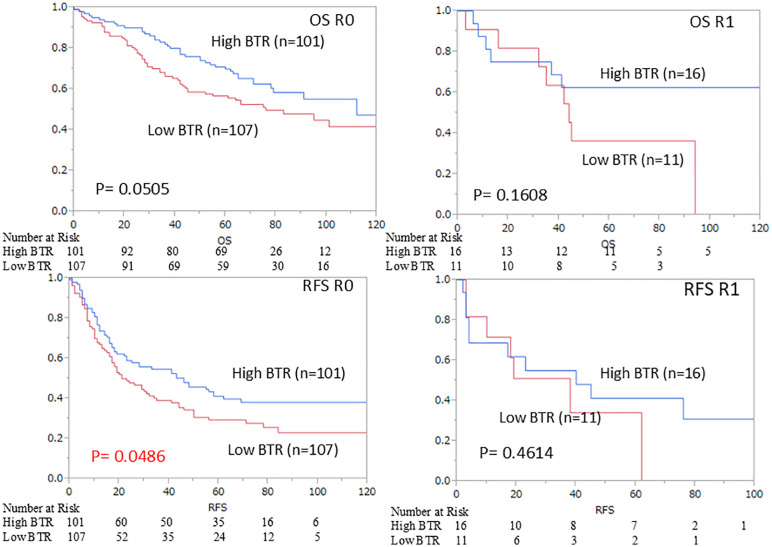
Kaplan–Meier curves for (A) overall survival (OS) of R0 group, (B) OS of R1 group,(C) recurrence‐free survival (RFS) of R0 group (D) RFS of R1 group.

The survival curves for OS and RFS are shown in Fig 3A and 3B, respectively. The median follow-up duration was 65 months. The median BTR was used as the cut-off value, dividing the population into high (117 patients) and low BTR groups (118 patients). Survival analyses, including OS and RFS for each group, are shown in Fig 3C and 3D. A significant survival benefit was observed in the high BTR group (p = 0.020). Hepatectomy involving the removal of two or more segments was defined as major hepatectomy. Statistically, a significant prolongation of survival was observed in the group with a high BTR in both overall survival (p = 0.045) and recurrence-free survival (p = 0.038), but only in the group with minor hepatectomy (Fig 4). And R0/1 was not a significant factor in either overall survival (p = 0.4837) or recurrence-free survival (p = 0.5389). However, when the groups were divided into R0 and R1, In the R0 group, high BTR was a significant factor associated with prolonged recurrence-free survival. In the R1 group, no correlation was found between BTR and OS (p = 0.1608) or RFS (p = 0.4614) (Fig 5).

### BTR and liver fibrosis based on histopathological background liver diagnosis, including comparison with ICG-R15

**Fig 6:**
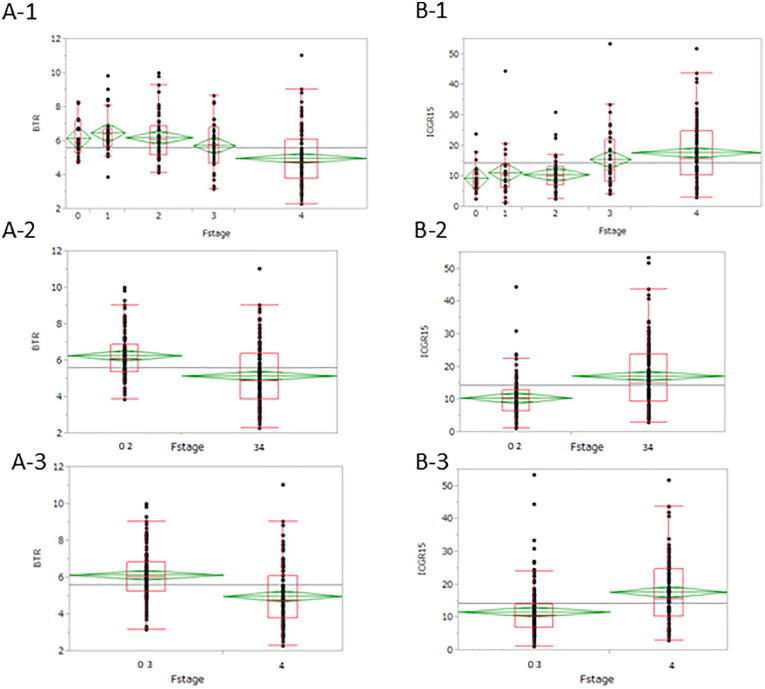
BTR and liver fibrosis based on histopathological background liver diagnosis, including comparison with ICG-R15. Both BTR and ICG-R15 showed significant variation in values depending on the degree of liver fibrosis (A-1, B-1). Both BTR and ICG-R15 showed significant differences in liver fibrosis when the F stage was divided into F0–2 and F3–4 (A-2, B-2). When divided by whether the patient had cirrhosis (F4), significant differences were also observed (A-3, A-4). BTR, branched-chain amino acid-to -tyrosine ratio; ICG-R15, 15-min retention rate of indocyanine green.

The correlation between fibrosis (F stage) of the background liver and preoperative BTR and ICG values in the resected specimens is shown in Fig 6. Significant differences were observed in both BTR and ICG values across the five fibrosis stages (F0 to F4). When the F stages were grouped into two categories (F0–2 and F3–4), BTR showed a strong and statistically significant difference, similar to that of ICG. This pattern was also evident when comparing groups with and without liver cirrhosis (LC; F4).

Receiver operating characteristic (ROC) curves for BTR and ICG were compared between the two groups. The area under the curve (AUC) in the two-group comparison between F0–2 and F3–4 was 0.7126 for BTR and 0.71361 for ICG. Similarly, the AUC for distinguishing LC (F4) was 0.72433 for BTR and 0.69631 for ICG.

### Summarize key findings

- BTR and 1/BTR strongly correlated with ICG levels.
- BTR had a significant correlation with ALBI.
- In the univariate analysis, BTR, ALB, intraoperative blood loss, and operative time were significantly correlated with postoperative complications. However, none of these factors were significant independent predictors in the multivariate analysis.
- A significant overall survival benefit was observed in the high BTR group. BTR and prognosis were evaluated according to resection margin status (R0 vs. R1) and whether the major hepatectomy. In cases of R1, BTR was not a significant predictive factor for either OS or RFS. On the other hand, in cases of R0, BTR was a significant prognostic factor for RFS. In cases of major hepatectomy, BTR was not a significant predictive factor for either OS or RFS. On the other hand, in cases of minor hepatectomy, BTR was a significant prognostic factor for both OS and RFS.
- Both BTR and ICG showed a correlation with pathological liver fibrosis. When considering whether or not cirrhosis was present, BTR was significantly more effective than ICG.

## Discussion

This study revealed that BTR and 1/BTR have a high correlation coefficient with ICGR-15 and a significant linear correlation. BTR is the ratio of BCAA (valine, isoleucine, and leucine) to tyrosine present in the plasma, serving as an alternative to Fischer’s ratio, which compares BCAA to AAA in the blood. It is widely recognized as a marker of reduced liver function [20]. ICG-R15, on the other hand, is the stagnation rate of intravenously injected ICG measured in real time at 15 min. The primary differences between these two tests are their simplicity and versatility. BTR is a routine blood test that does not require rest or specific timing and can be performed without a physician. Furthermore, it does not involve the use of reagents, eliminating the risk of intolerance or allergic reactions, and can be implemented immediately in any facility. The direct relationship between ICG and BTR metabolism is not yet clear. ICG indicates the pigment excretion capacity of the liver. The transporters involved in the absorption of this pigment excretion are Organic Anion Transporting Polypeptides (OATP) [21], while those involved in excretion are Multidrug Resistance Protein 3 (MRP3) and Multidrug Resistance-associated Protein 2 (MRP2) [22]. No reports were found regarding the direct relationship between BCAA and tyrosine and their transporters. However, an indirect relationship may potentially exist. While tyrosine is metabolized in the liver, BCAAs are metabolized primarily in the muscles and hardly at all in the liver. Both BCAAs and tyrosine utilize l-type amino acid transporter (LAT1) as their metabolic transporter. Reduced ICG retention rate associated with impaired liver function indicates decreased expression of OTAP and MRP. Decreased expression of transporters in the liver due to impaired liver function increases ICG retention rate and lowers BTR due to increased tyrosine. The Clinical Practice Guidelines for Hepatocellular Carcinoma in Japan strongly recommend preoperative ICG-R15 testing, and use the results and estimated resection volume, to determine surgical indications [10,11]. However, some patients have abnormal test results due to ICG excretory defects or jaundice, or they may be allergic to iodine and therefore unable to undergo the test.

Hepatobiliary scintigraphy using technetium-99m galactosyl serum albumin (^99m^Tc-GSA) is also listed in the guidelines, alongside ICG, as a test to assess liver reserve capacity [10,11]. However, this test is limited to facilities capable of conducting advanced medical imaging, and its evaluation is not standardized. Kawamura et al. conducted a correlation analysis between ICG and Tc-GSA, using either blood clearance or liver uptake values depending on the severity of liver injury. Their study, which included 282 cases, reported a correlation coefficient of 0.61 with p < 0.0001 [23]. Our study, involving a similar number of patients, showed that the correlation between 1/BTR and ICG was even stronger (r = 0.66, P < 0.001) in a simple correlation analysis. The ICGR15 prediction formula based on BTR derived in the present study may have significant clinical implications on hepatectomy in patients who cannot undergo ICGR15 testing. Regarding alternative liver function tests other than ICG and Tc-GSA, Hepatic venous pressure gradient (HVPG) and liver elastography exist. However, HVPG is an invasive test requiring transvenous catheter placement into the atrium and hepatic veins, and few facilities routinely perform it preoperatively. Liver elastography is mainly an ultrasound-based technique, but it has the limitation that the measurement technique itself can be influenced by factors such as respiration, the measurement site, and the degree of probe pressure applied [10].

And BTR was significantly correlated with the ALBI score, but not with the MELD score. These two scores evaluate liver reserve capacity, but they target different patient populations. The ALBI score has been reported to be useful as a predictor of long-term postoperative survival and liver failure.

The MELD score is a prognostic model originally developed to predict 3-month mortality in patients awaiting liver transplantation. Its applicability to prognostic assessment in HCC or in cases of mild liver cirrhosis has been questioned [24,25]. The result that BTR correlates more strongly with ALBI than with MELD supports its validity as a factor for assessing liver reserve capacity in clinical practice for patients with HCC. Furthermore, ALB is influenced by nutritional status and ALB transfusions. For patients with such factors clinically, BTR may be a more reliable method for assessing hepatic reserve capacity.

Low BTR was identified as a poor prognostic factor for OS. A similar trend was noted for RFS, although the difference was not statistically significant (p = 0.055). Several studies have reported an association between low BTR and poor prognosis in patients with liver cirrhosis [26]. In addition, some studies have indicated that low BTR is a poor prognostic factor for survival in patients with HCC [27,28]. BCAA have been reported to potentially play a role in suppressing HCC. The LOTUS trial, a multicenter randomized controlled study involving 646 patients with decompensated cirrhosis, demonstrated that BCAA administration delayed the onset of adverse events associated with cirrhosis progression (such as worsening hepatic decompensation, variceal bleeding, HCC development, and death) while improving survival and quality of life [29]. Furthermore, in a prospective multicenter study involving 299 cases including compensatory cirrhosis, the incidence of HCC was higher in the group without BCAA supplementation and also higher in the group with low BTR [30]. The key point in this mechanism is that BCAA play a role in regulating insulin resistance, and BCAA supplementation contributes to improving insulin resistance [31]. In mechanistic or experimental research, BCAA reported to decrease apoptosis inhibition and lower vascular endothelial growth factor, p-beta-catenin, p-p38 mitogen-activated protein kinase associated with HCC carcinogenesis [32].

The relationship between liver regeneration after resection and BCAA has also been reported. Portal vein embolization (PVE) is performed to induce hypertrophy of the remaining liver before hepatic lobectomy. Beppu et al reported that BCAA supplementation can improve postoperative functional liver regeneration of patients receiving [33].

When BTR and long-term prognosis were examined separately for major hepatectomy and minor hepatectomy, high BTR in minor hepatectomy contributed to overall survival and recurrence-free survival. Understanding these results is difficult. As a result, minor hepatectomy leads to fewer recurrences and a favorable prognosis if liver function impairment is minimal. HCC can develop lesions in other areas even after the target lesion is removed, a characteristic known as multicentric growth. In the high BTR group, this occurrence may be suppressed. On the other hand, in the group undergoing extensive liver resection, the target lesion is often already an advanced-stage cancer, and good liver function may not contribute to prognosis. And the relationship between BTR, OS, and RFS was clearly different depending on whether R0 or R1 was achieved. High BTR tended to prolong OS in the R0 group (p = 0.506), and RFS was found to have a statistically significant prolonging effect (p = 0.0486). We believe that the strong condition for high BTR to contribute significantly to prognosis is that the cancer has been completely resected.

Regarding poor prognosis in the high BTR group, 8 cases died within 1 years in the high BTR group. Interestingly, two cases were due to causes other than cancer (aspiration pneumonia and cerebral hemorrhage). The other six cases were all in the group that underwent extensive liver resection, and vascular invasion was confirmed by pathological diagnosis. The average tumor diameter was 9.1 cm. Of these six cases, recurrence was confirmed in five cases within four months. Two cases were R1 resection. On the other hand, only one case was diagnosed with cirrhosis (F4). In cases where the disease cannot be controlled by resection, it is difficult to predict the prognosis using BTR.

Based on the results of these subgroup analyses and the clinical backgrounds of patients who had a high BTR but nevertheless experienced poor outcomes, we consider that postoperative prognosis in patients with HCC is largely influenced by tumor-related factors, such as tumor progression and whether complete resection was achieved. In contrast, BTR reflects liver function, which affects the prognosis of all patients with HCC, and given the known role of BCAA as a suppressive factor for cancer progression [34,35], it is reasonable that BTR served as a prognostic indicator for overall survival in the entire cohort.

In our study, BTR was also significantly correlated with liver fibrosis in the background liver pathology of the resected specimens, with its sensitivity surpassing that of ICG. Liver decompensation leads to portal hypertension, which in turn causes the development of esophageal and gastric varices and splenorenal shunts. These changes result in an increase in circulating blood volume that bypasses the liver, leading to poor ICG values, even if the pathology has not progressed to cirrhosis.

In this study, BTR was not an independent predictor of postoperative complications or massive ascites. In both results, it was clear that surgical invasiveness (blood loss and operating time) was a greater factor than liver function. The reason for this result is that, despite the wide range of surgical invasiveness, such as blood loss and surgery time, the majority of patients (over 90%) were classified as Child A, and many had good liver function, making it difficult to show a significant difference in factors related to liver function, including BTR. In this study, most patients were classified as Child A. Even when including patients classified as Child B or C, BTR was found to have a significant negative correlation with the Child-Pugh score. Reports indicate that within these cohorts, over an average observation period of 5 years, the group with lower BTR had significantly higher rates of death and liver failure. The results of this study are consistent with these reports [26].

The present study has several limitations. First, it was a single-center retrospective study. Secondly, ICG intolerance was not assessed. Because this study was conducted in a resource‑limited clinical setting, postoperative follow-up measurement of BTR, a crucial factor for elucidating the relationship between BTR and postoperative outcomes, was not performed.

Our findings highlight that both tumor factors and liver function are critically important in predicting postoperative outcomes in HCC patients. However, to clarify how these two elements contribute across different patient populations and to what extent, further investigations in a more homogeneous cohort will be necessary.

We do not believe that BTR is more useful than ICG, as numerous studies on ICG and the postoperative outcomes of HCC have provided many years of evidence supporting its evaluation and safe surgical indications. However, BTR may be a valuable alternative for patients who are intolerant to ICG, in whom ICG testing may not accurately assess hepatic reserve, and in preoperative HCC patients with strong shunt contrast. And combining BTR with other clinical scores may improve the accuracy of predicting postoperative liver failure and long-term survival.

In modern times, several treatment options exist for HCC. Beyond liver resection, which offers the highest curative potential, other choices include RFA, TACE, chemotherapy, and liver transplantation. While liver resection provides high curative potential for patients with low hepatic reserve, it may also lead to postoperative liver failure or poorly controlled ascites, potentially causing postoperative mortality, prolonged hospitalization, and reduced quality of life. Based on the results of this study, we believe that BTR may contribute to individualized treatment approaches for HCC in the future by predicting the feasibility of safe liver resection.

## Conclusion

We showed that BTR is highly correlated with ICGR15, and that 1/BTR has an even stronger linear correlation with ICGR15, which are important factors in assessing preoperative liver function. For patients who cannot be accurately evaluated using ICG, BTR has been suggested to potentially play a crucial role in storing ICG. Furthermore, the predicted sensitivity of BTR for cirrhosis (stage 4) was higher than that of ICGR15. The result is This is a clinically significant finding that substantiates its usefulness as a liver reserve capacity assessment factor. BTR is also a long-term prognostic factor for HCC patients, it sufficiently suggests the potential for integration into predictive models for the precise management of HCC.

This study is a retrospective single-center study. To improve the accuracy of ICG-based predictive models incorporating BTR, and to evaluate the utility of BTR as a prognostic factor following hepatic resection, prospective multi-center studies are needed.

## Supporting information

S1 Data Dataset. Minimal dataset of this study.
(XLSX)
